# Acute Radial Compressive Neuropathy: The Most Common Injury Induced by Japanese Rope Bondage

**DOI:** 10.7759/cureus.39588

**Published:** 2023-05-28

**Authors:** Vasily Khodulev, Artsiom Klimko, Nataliya Charnenka, Marina Zharko, Hanna Khoduleva

**Affiliations:** 1 Department of Functional Diagnostics, Republican Research and Clinical Center of Neurology and Neurosurgery, Minsk, BLR; 2 Department of Neurology, University Hospital of Zurich, Zurich, CHE; 3 Department of Diagnostic Sonography, Multidisciplinary Medical Center "Healthy Sleep Center", Minsk, BLR; 4 Department of Anatomical Pathology, City Clinical Pathologoanatomic Bureau, Minsk, BLR; 5 Department of Pediatrics, Belarusian State Medical University, Minsk, BLR

**Keywords:** compression neuropathy, ultrasound examination, conduction block, nerve conduction studies, kinbaku, shibari, japanese rope bondage

## Abstract

Japanese rope bondage (RB), or Shibari, is an art form involving the voluntary and aesthetic binding of a person with a rope, which may result in compression injuries to peripheral nerves. To investigate the nature and extent of nerve injuries associated with this practice, we conducted a survey of four experienced RB practitioners (riggers) and participants who were willing to share their experiences of injury. Injuries presented acutely and immediately following full-body suspensions, with a total of 10 individuals (16 injuries) identified with damage to the radial, axillary, or femoral nerves. Notably, the radial nerve was the most commonly affected structure in our patient cohort, with 90.0% of individuals experiencing an injury at this level.

We present a rare case of acute repeated compression of the radial nerve during full-body suspension RB. A 29-year-old female was suspended for 25 minutes using a 6-mm jute rope, resulting in wrist and finger drop, as well as reduced sensation in the left hand. Analysis revealed a 77.3% conduction block in the upper arm segment. Improvement was observed after three months, fully achieved after five months. Seventeen months later, re-compression of both radial nerves occurred during a similar suspension lasting 8-10 minutes. Improvement occurred after one week, fully achieved after four weeks. The third compression episode occurred three years later, lasting five minutes, with full recovery within two minutes. This study focuses on the injury of peripheral nerves, including the radial, axillary, and femoral nerves, namely, acute compression neuropathy induced by Japanese RB. Because the radial nerve is the most frequently injured structure, the findings underscore the significance of recognizing the anatomical course of the radial nerve, particularly its position posteriorly at the distal deltoid tuberosity level, as a means of preventing nerve injury in this region. This knowledge is particularly crucial for individuals engaged in the practice of RB, emphasizing the importance of taking precautions to avoid potential nerve damage.

## Introduction

Rope bondage (RB), also known as Shibari, is a practice that has evolved over centuries in Japan, encompassing both combat and erotic techniques [1,2]. It involves one partner restricting the physical mobility and/or freedom of action of another partner to varying degrees for psychosexual and/or aesthetic gratification. Despite its growing popularity in recent years, RB remains marginalized due to negative connotations. Jones explored the diverse perspectives of participants in RB, focusing on personal play and the social world [2]. Motives and emotions surrounding RB vary widely among participants, from exploring bodily sensations to mastering tying techniques and appreciating the aesthetic aspects of the practice. However, RB carries potential hazards, including peripheral nerve damage resulting in motor and sensory impairments and, in severe cases, fatalities. Inversions during full-body suspension increase the risk of falls, head injuries, and asphyxiation. Despite warnings about peripheral nerve damage, scientific literature lacks sufficient information on the prevalence and specifics of nerve injuries caused by RB.

RB involves half-suspension and full-suspension, with the latter posing a higher risk for compression injuries to peripheral nerves. This article aims to address this gap in knowledge by providing a detailed report of nerve injuries in RB, with a focus on identifying the nerves most vulnerable to acute compression neuropathy. Our findings supplement a previously reported clinical nerve conduction study (NCS) case report involving acute, repeated compression of the radial nerve during the suspension, which was presented as a poster at the 17th European Congress of Clinical Neurophysiology in 2019 [3]. Incorporating data from our survey, we provide additional information and insight into the nature and frequency of nerve injuries associated with RB, which can inform efforts to prevent such injuries.

## Materials and methods

To address the dearth of knowledge on nerve injuries related to RB, we conducted a retrospective survey among four experienced RB practitioners and participants who shared their experiences with injuries that occurred during RB. The participants were recruited through online forums and social media platforms for RB enthusiasts, as well as through the personal networks of the practitioners. Through semi-structured interviews, we sought to gain insight into their experience with RB, their understanding of nerve anatomy and injury, the types of binding techniques they use, and the precautions they take to prevent nerve injuries.

During the semi-structured interviews, participants were asked a range of open-ended questions to gather information on their demographics, medical history, RB experience, injury characteristics, and recovery outcomes. Questions such as "Can you tell me a bit about yourself?" were used to gather demographic information, including age, sex, and occupation. Participants were also asked about their experience in RB, such as "How long have you been practicing rope bondage, and how frequently do you engage in it?" To assess potential influencing factors, questions like "Have you ever had any previous medical conditions or injuries that you think may have influenced your risk of nerve injuries during rope bondage?" were posed. Additionally, participants were encouraged to describe their specific nerve injuries, including symptoms and manifestations, such as "Could you describe the specific nerve injury you experienced during rope bondage? How did it manifest, and what were the symptoms?" The interviews also aimed to capture recovery outcomes, with questions like "How long did it take for you to notice improvements in limb movement after the nerve injury?" Furthermore, participants were asked about seeking medical attention, modifications to their practices, and long-term effects, such as "Did you seek medical attention or consult a healthcare professional regarding your nerve injury? If so, how did they assist you in your recovery?" and "Have you noticed any long-term effects or residual symptoms from the nerve injury?"

All four practitioners had three to five years of binding practice and handled approximately 100 binding cases per year. We also asked them about their experiences with clients who had suffered nerve injuries and how they addressed these injuries. We identified 10 individuals (16 injuries) who suffered peripheral nerve damage leading to motor impairment after RB suspension. We collected demographic and anthropometric data on each participant, including age, sex, height, weight, and body mass index. A semi-structured survey was designed to collect information on the participants' demographics, anthropometric data, previous medical history, RB experience, injury characteristics, and recovery outcomes. The survey consisted of open-ended questions, as well as multiple-choice and Likert scale questions. The sample size was not predetermined as this was an exploratory study, and there was limited data available on the topic. We aimed to collect as much data as possible from willing participants to gain a comprehensive understanding of nerve injuries associated with RB.

Through this survey, we collected subjective information on the affected nerves, the duration of the suspension, recovery time for limb movement, and whether or not medical attention was sought. We recorded the type of binding techniques used, the position of the rope on the body, and any potential contributing factors leading up to the injury. Additionally, we gathered information on any previous nerve injuries or medical conditions that may have contributed to the injury. Nerve injuries were classified according to the type and location of the affected nerve. We also recorded the duration of the nerve compression or traction injury. Data was collected through online surveys and interviews, and the information collected was analyzed to identify common themes and patterns. All participants provided informed consent before participating in the survey. The study has several limitations, including the small sample size and the potential for selection bias due to the recruitment methods used. The retrospective nature of the study also makes it susceptible to recall bias. Additionally, the survey only included individuals who had experienced nerve injuries, and therefore may not provide a complete understanding of the risks associated with RB.

## Results

In our research, with the exception of a singular case, all nerve injuries transpired acutely and promptly following full-body suspension. Through our survey, we have identified 10 individuals who suffered peripheral nerve damage leading to motor impairment, with a total of 16 injuries, along with one participant who developed bilateral proximal (supraclavicular) brachial plexopathy a few days after suspension. Furthermore, some participants encountered fleeting paresthesia and numbness in their hands and fingers when the rope was positioned or shifted around the wrist area; however, these occurrences were not included in our study. Table 1 provides details on demographic and anthropometric data, as well as information on the affected nerves. Two patients experienced multiple injuries, and one participant endured simultaneous compression injuries to the nerves of both upper and lower limbs. Bilateral damage to the same nervous structures was evident in two cases, involving the radial and femoral nerves.

**Table 1:** Demographic and anthropometric data, along with the affected nerves and the duration of suspension and time to recovery of sensation and movement BMI: body mass index; *: the same person; †: this case is not included in the main cohort; R: right; L: left; NA: not applicable (missing data); M: male; F: female

Case number	Age (years)	Sex	BMI (kg/m^2^)	Affected nerve	Suspension time	Time to recovery of sensation and movement
1а*	29	F	17.5	Radial L	25 min	5 months
1b*	30	F	17.5	Radial bilateral	8-10 min	4 weeks
1c*	33	F	17.5	Radial L	5 min	2 min
2	31	F	21.2	Radial R	10–15 min	3 months
3a*	29	F	NA	Radial L	10 min	within 1 day
3b*	29	F	NA	Radial R	10 min	within 1 day
4	20	F	19.9	Radial L and bilateral femoral	15 min	10 days
5	35	M	24.5	Radial R	5 min	within 1 day
6	27	F	26.0	Radial R	5–10 min	within 1 day
7	21	M	26.1	Radial L	10 min	3 hours
8	19	F	19.9	Radial R	30 min	20 days
9	35	F	19.5	Radial L	NA	2-3 weeks
10	30	M	21.2	Axillary R	30 min + mild shoulder pressure	1 month
11†	28	F	22.9	Bilateral proximal brachial plexopathy	Several seconds	1 month

**Figure 1:**
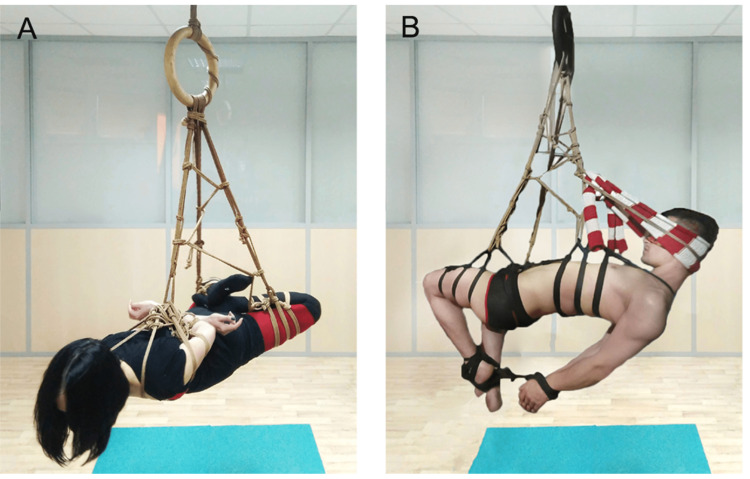
The figures illustrate a full horizontal suspension (A) with damage to the radial nerve in the middle third of the upper arm (case 1) and (B) injury to the axillary nerve (case 10)

Among the affected individuals, five sought medical attention. NCSs were performed on two patients (cases 1 and 2). Our analysis demonstrated that the radial nerve at the upper arm was the most commonly affected structure in our patient cohort, with 90.0% of patients and 81.3% of injuries involving this nerve. Typically, the rope was situated in the middle third of the upper arm to facilitate a horizontal or lateral suspension, with the individual's body facing downward or sideways, respectively. The hands were frequently tied in a box-tie configuration (as illustrated in Figure 1A). Affected individuals frequently reported experiencing numbness of the dorsal surface of the wrist and varying degrees of weakness in wrist and finger extensor muscles, ranging from complete paralysis to moderate weakness. Furthermore, sensory loss was observed on the lateral dorsum of the hand.

In one instance, an isolated compression injury of the axillary nerve arose due to suspension from the torso, with the individual lying face-up, and the hands tied at the wrist, hanging down under the influence of gravity (Figure 1B). After 30 minutes, a slight pressure was exerted on the shoulders, which triggered a sudden electric shock-like pain and presyncopal state. The participant was expeditiously removed from the suspension, following which he was incapable of abducting the right arm. In another case (case 4), concurrent injuries to the femoral and radial nerves were observed, resulting in weakness in wrist extension and sensory disturbances to the anterior surface of the thigh, along with a sense of instability around the knee. As a consequence, binding around the thigh consistently elicited sensory disturbances on the anterior surface of the thigh in this female participant.

A notable feature of case 11 is the development of bilateral proximal brachial plexopathy several days after suspension, which is why we did not include this case in the main group. During the suspension, a sudden lateral jerk and body rotation caused intense pain in the upper limbs, which subsided after a few minutes. The exact positioning of the arms is unknown, but it is known that there were few rope turns, they were poorly distributed on the body, and the binding slipped during the session. The following day, the patient reported experiencing pain in the clavicular region, which was subsequently followed by weakness in the proximal regions of both arms, rendering her incapable of abduction, lifting, and flexing at the elbow several days later. The patient did not seek medical attention, and strength was restored spontaneously after one month.

Individuals who engage in Japanese RB do so for diverse reasons and experience a range of emotional responses. Some participants seek to surrender themselves and trust their partner, while others desire introspection and solitude. Sensory exploration is also common, with some seeking to achieve a trance-like state or a sense of calm, while others engage in the practice of sexualized actions and psychological pleasure. Additionally, participants may be motivated by curiosity, novelty, or aesthetic pleasure. During the process, individuals may experience euphoria and focus on their sensations rather than the end result. Practitioners utilize a variety of communication methods, such as non-verbal communication and the use of ropes, knots, and tying options, to provide a sense of control and power over the participant and to achieve aesthetic and erotic satisfaction. By understanding the diverse range of motives and emotional responses of individuals who engage in Japanese RB, practitioners can better communicate with participants about the potential risks and dangers of nerve injuries, as well as provide guidance on how to avoid such injuries. Raising awareness of the potential for nerve damage and how to prevent it can help to promote the safe and responsible practice of this cultural tradition.

Case report of acute repeated compression of the radial nerve caused by full-body suspension

We present an atypical instance of acute repeated compression of the radial nerve that occurred during RB full-body suspension. The affected individual was a 29-year-old female with a body mass index of 17.5, who was suspended by the upper limbs, torso, and hips for 25 minutes, using a 6-mm jute rope placed in the middle third of the upper arm (Figure 1A). Within Table 1, this patient corresponds to cases 1A, 1B, 1C, 3A, and 3B. After the removal of the rope, she experienced wrist and finger drop in the left hand, along with reduced sensation in the posterolateral hand. Initially, medical attention was not sought, but eventually, on the 48th day after the RB session, the individual sought medical care at our department. Neurological examination revealed weakness in finger and wrist extension, numbness in the dorsal surface of the hand innervated by the radial nerve, and a reduced carporadial reflex.

**Figure 2:**
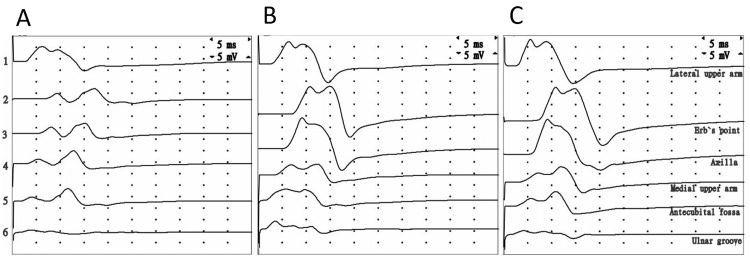
Recording of CMAPs from the extensor digitorum muscle at six different points of stimulation, arranged top to bottom: (1) the distal part of the lateral upper arm, (2) Erb's point, (3) the axilla, (4) the middle part of the medial upper arm (median and ulnar nerves), (5) the antecubital fossa (median nerve only), and (6) the ulnar groove (ulnar nerve only). The recordings were taken at three time points: (A) 48 days after the initial nerve damage, (B) three years after full recovery, and (C) on the unaffected side

Radial NCSs were conducted using the technique described previously [4]. The compound muscle action potential (CMAP) was elicited from the extensor digitorum muscle by stimulating six different points (Figure 2A). The points of stimulation were as follows: (S1) the distal point, located 6 cm proximal to the lateral epicondyle of the humerus (the radial nerve), (S2) Erb's point (at the level of the brachial plexus), (S3) the apex of the axilla (the radial, median and ulnar nerves), (S4) the middle part of the medial section of the upper arm (the median + ulnar nerves), (S5) the antecubital fossa (the median nerve), and (S6) the elbow area below the ulnar groove (the ulnar nerve). To determine conduction block (CB), three main stimulation points are required: distal, proximal (Erb's point or the axilla), and median+ulnar points. The remaining points were included to evaluate the formation of the CMAP in the extensor digitorum muscle during brachial plexus stimulation. The total distal CMAP area was calculated by adding the distal and median+ulnar CMAP areas together. The CB was calculated by determining the difference between the total distal CMAP area and Erb's CMAP area as a percentage, using the following formula: (total distal CMAP area - Erb's CMAP area) × 100/(total distal CMAP area).

The results of the motor NCSs are presented in Table 2 and Figure 2. We observed a CB of 77.3% at the upper arm segment, accompanied by a slight reduction in distal CMAP amplitude, although no decrease in conduction velocity was noted. Additionally, a reduction in median, ulnar, and median+ulnar CMAP areas was observed on the affected side in comparison to the unaffected side and the same side after full recovery (as illustrated in Figure 2B, 2C). Sensory NCSs did not yield anything of interest (normal results). Clinical improvement commenced after three months and was completely achieved after five months. Seventeen months after recovery, the patient experienced re-compression of both radial nerves during a similar suspension lasting approximately 8-10 minutes. Improvement began after one week and was fully attained after four weeks. The third compression episode occurred three years after the first episode and lasted five minutes, with complete clinical recovery occurring in two minutes. NCSs performed one month after the third compression episode demonstrated normal motor and sensory conduction in the radial nerve (Table 2, Figure 2B), as well as in the median, ulnar, fibular, tibial, and sural nerves on both sides.

**Table 2:** The results of the radial motor NCSs CMAP: compound muscle action potential; CB: conduction block; *: conduction velocity was calculated via the distal latency between the distal points and extensor digitorum (conduction velocity times latency)/between the distal point and Erb's point

NCS parameters	Affected side		Unaffected side
	48 days after injury	Three years after injury	
1. Distal CMAP area (mVms)	40.1	58.7	67.9
2. Erb`s CMAP area (mVms)	23.3	84.2	101.0
3. Axilla CMAP area (mVms)	22.2	76.1	86.0
4. Median + ulnar CMAP area (mVms)	22.2	31.0	37.7
5. Median CMAP area (mVms)	20.6	34.3	32.2
6. Ulnar CMAP area (mVms)	4.6	11.7	8.1
Total distal CMAP area (mVms)	62.3	89.7	105.6
CB, area (%)	77.3	6.1	4.4
Conduction velocity (m/s)	50.0/66.0*	52.0/69.0*	48/70.0*
Distal CMAP latency (ms)	2.7	2.6	2.5
Axilla CMAP latency (ms)	5.5	5.7	5.6
Erb`s CMAP latency (ms)	6.9	6.8	6.8
Distal/Erb`s CMAP duration (ms)	10.3/12.4	9.8/10.5	9.5/11.1

**Figure 3:**
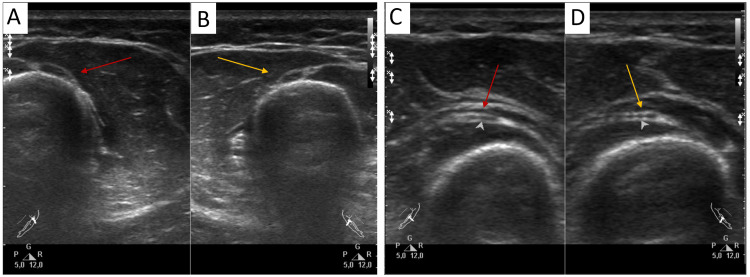
The presented transverse sonograms depict the affected (left, red arrow) and unaffected (right, orange arrow) sides of the radial nerve at the spiral groove (A, B) and the deep branch of the radial nerve at the supinator muscle (C, D). A high-resolution ultrasound examination revealed a mild enlargement in the cross-sectional area of the radial nerve on the affected side at the spiral groove only (A), compared to the contralateral side

High-resolution ultrasound examination revealed the radial nerve on the affected side to have a clear, smooth contour, a heterogeneous fascicular internal structure, and normal echogenicity (Figure 3A, 3B). The cross-sectional area of the affected nerve was within the normal range, measuring 0.10 cm^2^ (reference range: 0.065-0.116 cm^2^). Notably, this value was higher than the mean values (0.087 ± 0.009 cm^2^) and larger than the cross-sectional area of the nerve on the opposite, unaffected side (0.081 cm^2^). At the elbow joint level, there were no differences in cross-sectional area between both nerves (Figure 3C, 3D).

## Discussion

It is crucial to recognize that RB carries potential hazards, particularly when performed without proper knowledge, skills, and precautions. It may result in peripheral nerve damage, leading to both motor and sensory impairments and, in severe cases, even fatalities. Despite numerous warnings about the possibility of peripheral nerve damage, there remains a scarcity of comprehensive information within the scientific literature regarding the prevalence and detailed specifics of nerve damage caused by RB. Despite its growing popularity, RB remains marginalized due to its negative connotations [5]. Although we cannot speculate on the overall incidence of nerve damage in the broader RB community, our study findings clearly demonstrate that the risk of nerve injuries associated with RB is not negligible. The prevalence of nerve damage observed in our surveyed participants underscores the importance of recognizing and addressing these risks.

Our study revealed that the most common type of nerve damage occurred to the radial nerve at the level of the spiral groove, which corresponds to the mid-humerus region, and had varying degrees of severity. This type of nerve damage accounted for 81.3% of all nerve injuries (13 out of 16 injuries) or 90.0% of individuals (9 out of 10 individuals). While temporary numbness and paresthesia in the hand were frequently reported, participants did not attribute much significance to them, as they did not lead to persistent sensory or motor impairments. In such cases, the median and ulnar nerves, as well as the superficial sensory branch of the radial nerve, which are particularly susceptible at this anatomical level, may also be affected. A prime example of this type of injury is handcuff-related nerve damage attributed to overtightened handcuffs. In these cases, the most frequent and severe damage was observed in the radial nerve, either unilaterally or bilaterally [6]. The superficial sensory branch of the radial nerve is highly susceptible to direct traumatic injury. It lies close to the dorsolateral aspect of the radius near the wrist and its superficial location makes it vulnerable to peripheral compressive forces such as a tight watchband, handcuffs, or rope.

Radial neuropathy at the spiral groove is the third most frequent compressive neuropathy syndrome of peripheral nerves of the upper limb encountered in primary care settings [7]. However, the frequency of radial neuropathies in the context of acute compression neuropathies with CB remains unclear. Our study revealed a range of nerve damage patterns, including unilateral or bilateral injuries, single or multiple nerve compressions, and durations of compression with the rope lasting from approximately 5 to 25 minutes. The recovery time for motor or sensory disturbances varied from two minutes to five months. Radial nerve injuries typically occur due to compression at the level of the mid-humerus. Approximately 6.3 cm of the radial nerve is in direct contact with the posterior humerus, centered over the distal aspect of the deltoid tuberosity [8]. This anatomical feature may explain the observed injury patterns in our study. Indeed, external compression of the radial nerve in the setting of crutch use, improper sleeping positions, tourniquet compression, and compromised intraoperative positioning have all been reported [9]. We did not find any reports delineating radial nerve injuries to compression by RB.

The underlying mechanism behind acute compressive radial neuropathy is attributed to focal demyelination produced by mechanical compression. However, animal experiments have shown that low-magnitude extraneural compression can lead to decreased intraneural microvascular flow, impaired axonal transport, and alterations in nerve structure and function [10]. Continued pressure precipitates Schwann cell damage and myelin displacement; electrophysiologically this manifest as CB, which reflects focal demyelination of motor axons [11]. In rats with extraneural compression applied to the sciatic nerve, demyelination was prominent in the nerves subjected to 4.0 kilopascals of compression, while axonal degeneration was observed in the nerves subjected to 10.7 kilopascals of compression [12]. Axonal degeneration results in a reduction in the amplitude and area of the CMAP due to a decrease in axonal number. NCSs can be used to evaluate radial motor nerve fibers, although they are more complex than those used for median and ulnar nerves [4,13].

The first case in our patient cohort is the most striking, and, therefore, we will focus the discussion on this patient. For this patient, the initial episode of radial nerve compression appeared to correspond to both focal demyelination and axonal degeneration. The presence of a CB confirmed the presence of focal demyelination. The normal conduction velocity values observed in the upper arm are attributed to the activity of the median and ulnar nerves upon brachial plexus stimulation [4]. Axonal degeneration was confirmed both electrophysiologically and clinically in our study. Electrophysiologically, a reduction in the amplitude and area of the distal motor response was observed in comparison to the unaffected side, and recovery of the distal CMAP was observed after complete recovery. Clinically, the duration of nerve function recovery was tracked and lasted for five months. Typically, recovery of radial nerve function resulting from focal demyelination occurs within approximately 8-12 weeks after the onset of symptoms [14]. However, in cases of axonal damage resulting from collateral sprouting and axonal regrowth, recovery may be prolonged.

Another feature of axonal degeneration observed in this case was a decrease in the median and median+ulnar CMAP areas compared to those of the unaffected side and affected side after recovery (as seen in Figure 2 and Table 2), similar to what was observed in traumatic radial neuropathy [4]. This phenomenon can be attributed to the structural changes that occur in the muscles innervated by the radial nerve, leading to partial attenuation of the volume-conducted signal from the intact anterior compartment of the forearm muscles. Studies have indicated that the presence of collagen fibers and fatty degeneration in muscle tissue can cause alterations in the reflection of ultrasound beams and X-rays [15,16]. In cases of recurrent focal pressure neuropathies, the possibility of hereditary neuropathy with increased susceptibility to pressure palsies should also be considered. However, in our case, there were no signs of symptomatic or asymptomatic mild polyneuropathy or evidence of previous nerve other palsies, as there was an absence of focal weakness, atrophy or sensory loss, or lack of ankle reflexes.

The minimum amount of time necessary for the development of compressive neuropathy is unclear. Animal models have shown that a persistent injury to the posterior tibial nerve can be caused by applying a tourniquet for as little as one hour [17]. In humans, this may be even shorter, as studies have described paresis of the foot appearing during fibular nerve compression in as little as 25 minutes [18]. We previously identified two cases where compressive radial neuropathy appeared during 20 to 30 minutes of sleep [4], and another case was reported where paresis of the hand extensor developed after leaving the arm hanging over the armrest of a chair for 30 minutes [19]. The minimum time for the onset of acute numbness or weakness associated with handcuffs was 15 minutes [6]. In case 1, the patient with repeated injuries experienced compression times of 25, 8, and 5 minutes, respectively, with a clinical recovery time of 5 months, 4 weeks, and 2 minutes. Animal experiments have shown that remyelination occurs faster in cases of nerve compression with a narrow rope compared to a broader compressive cuff [20]. Hence, the duration of the injury and the number of affected Ranvier nodes are important factors to consider as they can impact the prognosis of the condition. The type of myelin damage also impacts recovery time and could be classified as a node of Ranvier displacement, partial or complete breakdown of the stretched paranodal myelin, and intra- or periaxonal edema [18,21].

Patient 4 reported symptoms consistent with femoral neuropathy, including a sensory loss in the anterior thigh and a sense of instability around the knee. Femoral neuropathy is typically characterized by weakness in knee extension and hip flexion, along with sensory loss in the anterior and medial thigh as well as the medial lower leg [22]. This condition is commonly caused by compression at the inguinal ligament when the hip is flexed and externally rotated and classically occurs when patients are placed in the lithotomy position for prolonged periods of time during surgical procedures [23]. Iatrogenic injury during intra-abdominal and pelvic surgical procedures, as well as gynecologic and urologic interventions, are the most leading causes of femoral neuropathy [24]. It is likely that the rope caused compression of the femoral nerve under the inguinal ligament in this case.

Axillary nerve injury is commonly associated with shoulder trauma, which can be caused by direct compression or traction on the nerve. This can occur in a variety of situations, including shoulder surgery and motor-vehicle accidents. Other causes include pressure from crutches or hyperextension of the shoulder, as may occur in some contact sports [11]. In case 10, compression or traction on the nerve occurred due to hyperextension of the participant's shoulders during RB. Compression or traction injuries to the nerve typically result in CB without structural changes and symptoms that disappear within a few weeks [25].

Cervical roots of the brachial plexus are most frequently injured, often due to closed traction. Burner syndrome, rucksack paralysis, and classic postoperative paralysis are examples of brachial plexopathies that commonly affect the upper plexus. Burner syndrome occurs when sudden, forceful shoulder contact causes lateral flexion of the head in tandem with shoulder depression. Patients with upper plexopathies tend to have better recovery rates, as these lesions are typically caused by demyelinating CB, which is located closer to the muscles they innervate [26]. In case 11, it is unclear what caused the muscle weakness and whether there was a causal relationship with RB.

Lastly, preventive measures that can be implemented to avoid neurological damage during RB practices must be discussed. These measures include several overarching principles. Firstly, using techniques that distribute pressure evenly and avoid excessive compression on specific nerve pathways, such as the radial nerve. This may involve learning different knotting styles and positions that reduce excessive pressure on critical neuroanatomical areas. Limiting the duration of full-body suspensions to prevent prolonged compression on nerves. Secondly, setting time limits and regularly checking in with participants to ensure they are not experiencing excessive pressure or discomfort. Thirdly, ensuring proper positioning of the body and limbs during RB to minimize tension in vulnerable areas. Avoiding extreme angles or positions that put excessive strain on nerves can help prevent nerve compression. Fourthly, establishing open communication between practitioners and participants to allow for prompt identification of any signs or symptoms of nerve compression. Encouraging participants to communicate any discomfort or unusual sensations during the session can help catch potential issues early on. Lastly, providing comprehensive education and training on anatomy, nerve pathways, and safety guidelines for RB practitioners. This includes understanding the specific vulnerabilities of certain nerves and how to minimize the risk of damage during practice. Implementation of these preventive measures may enhance participant safety and minimize the risk of neurological damage during RB. However, we readily concede that further research and expert consensus is needed to develop comprehensive and standardized safety guidelines for RB practices.

It is important to note that this study was conducted in an exploratory manner, and the small sample size and non-randomized recruitment methods limit the generalizability of the findings. Nevertheless, our study provides valuable insights into the nature and extent of nerve injuries associated with RB and highlights the need for further research on this topic. Future studies could employ larger sample sizes, randomized recruitment methods, and more rigorous study designs to provide a more comprehensive understanding of the risks associated with RB and nerve injury.

## Conclusions

In the current study, we demonstrate injury of the radial, axillary, and femoral nerves in the form of acute compression neuropathy caused by Japanese RB. The radial nerve was the most frequently affected structure. The deltoid tuberosity is a reliable and practical anatomical landmark that can be utilized to determine the level of the radial nerve along the posterior aspect of the humerus not only during the surgery but also during Japanese RB. Knowledge of the direct posterior location of the radial nerve at the level of the distal deltoid tuberosity may help prevent nerve injury in the region of the readily palpable deltoid tuberosity. Recovery from RB-related nerve compression injuries varies depending on the severity and duration of compression, individual factors, and prompt medical intervention. In our study, most participants experienced improvements in motor function and sensation over time. Seeking timely medical attention and implementing safety measures are important for optimizing recovery outcomes. However, there is a risk of recurrent injuries if precautions are not taken. Overall, the prognosis for recovery after nerve compression in RB-related injuries is generally favorable, but practitioners and participants should prioritize safety, communication, and awareness of potential risks to prevent further nerve damage and promote optimal recovery.

## References

[1] Midori Midori (2001). The seductive art of Japanese bondage. Greenery Press.

[2] Jones Z (2020). Pleasure, community, and marginalization in rope bondage: a qualitative investigation into a BDSM subculture. Ontario: Carleton University.

[3] Khodulev V, Zharko M, Vlasava S, Charnenka N (2019). P17-S Repeated compression radial neuropathies caused by rope bondage. Clin Neurophysiol.

[4] Khodulev VI, Nechipurenko NI, Shcharbina NY (2020). Radial motor nerve conduction studies in the upper arm. Muscle Nerve.

[5] Pennington H (2017). Kinbaku: the liminal and the liminoid in ritual performance. Performance of the Real e-Journal [Internet.

[6] Grant AC, Cook AA (2000). A prospective study of handcuff neuropathies. Muscle Nerve.

[7] Latinovic R, Gulliford MC, Hughes RA (2006). Incidence of common compressive neuropathies in primary care. J Neurol Neurosurg Psychiatry.

[8] Carlan D, Pratt J, Patterson JM, Weiland AJ, Boyer MI, Gelberman RH (2007). The radial nerve in the brachium: an anatomic study in human cadavers. J Hand Surg Am.

[9] Markiewitz AD, Merryman J (2005). Radial nerve compression in the upper extremity. J Hand Surg Am.

[10] Rempel DM, Diao E (2004). Entrapment neuropathies: pathophysiology and pathogenesis. J Electromyogr Kinesiol.

[11] Kimura J (2013). Electrodiagnosis in diseases of nerve and muscle: Principles and practice. https://books.google.ch/books?hl=en&lr=&id=h06nBAAAQBAJ&oi=fnd&pg=PP1&dq=Electrodiagnosis+in+diseases+of+nerve+and+muscle:+Principles+and+practice+/+Jun+Kimura&ots=gSC0Ol-K7w&sig=CektHWGlZsukj3vxT6XsdZ6OMsY&redir_esc=y#v=onepage&q=Electrodiagnosis%20in%20diseases%20of%20nerve%20and%20muscle%3A%20Principles%20and%20practice%20%2F%20Jun%20Kimura&f=false.

[12] Powell HC, Myers RR (1986). Pathology of experimental nerve compression. Lab Invest.

[13] Dumitru D, Amato A, Zwarts M (1233). Electrodiagnostic medicine. https://www.elsevier.com/books/electrodiagnostic-medicine/dumitru/978-1-56053-433-4.

[14] Alin G, Matthew V (2023). Radial nerve injury. https://www.ncbi.nlm.nih.gov/books/NBK537304/.

[15] Hu CF, Chen CP, Tsai WC, Hu LL, Hsu CC, Tseng ST, Shau YW (2012). Quantification of skeletal muscle fibrosis at different healing stages using sonography: a morphologic and histologic study in an animal model. J Ultrasound Med.

[16] Goodpaster BH, Kelley DE, Thaete FL, He J, Ross R (2000). Skeletal muscle attenuation determined by computed tomography is associated with skeletal muscle lipid content. J Appl Physiol (1985).

[17] Fowler TJ, Danta G, Gilliatt RW (1972). Recovery of nerve conduction after a pneumatic tourniquet: observations on the hind-limb of the baboon. J Neurol Neurosurg Psychiatry.

[18] Garth MB (2015). Peripheral nerve disorders: a practical approach. https://www.cambridge.org/core/journals/canadian-journal-of-neurological-sciences/article/peripheral-nerve-disorders-a-practical-approach-1984-edited-by-ak-asbury-and-rw-gilliatt-published-by-butterworth-and-co-ltd-339-pages/F0E25736EAA562155B38D07034BE5634.

[19] Kim KH, Park KD, Chung PW (2015). The usefulness of proximal radial motor conduction in acute compressive radial neuropathy. J Clin Neurol.

[20] Rudge P, Ochoa J, Gilliatt RW (1974). Acute peripheral nerve compression in the baboon. J Neurol Sci.

[21] Ochoa J, Fowler TJ, Gilliatt RW (1972). Anatomical changes in peripheral nerves compressed by a pneumatic tourniquet. J Anat.

[22] Bowley MP, Doughty CT (2019). Entrapment neuropathies of the lower extremity. Med Clin North Am.

[23] David CP, Barbara ES (2021). Electromyography and neuromuscular disorders: clinical-electrophysiologic-ultrasound correlations.

[24] Craig A (2013). Entrapment neuropathies of the lower extremity. PM R.

[25] Mitchell JJ, Chen C, Liechti DJ, Heare A, Chahla J, Bravman JT (2017). Axillary nerve palsy and deltoid muscle atony. JBJS Rev.

[26] Ferrante MA (2004). Brachial plexopathies: classification, causes, and consequences. Muscle Nerve.

